# Is Transcranial Sonography Useful to Distinguish Scans Without Evidence of Dopaminergic Deficit Patients From Parkinson's Disease?

**DOI:** 10.1002/mds.25102

**Published:** 2012-06-28

**Authors:** Heike Stockner, Petra Schwingenschuh, Atbin Djamshidian, Laura Silveira-Moriyama, Petra Katschnig, Klaus Seppi, John Dickson, Mark J Edwards, Andrew J Lees, Werner Poewe, Kailash P Bhatia

**Affiliations:** 1 Department of Neurology, Medical University InnsbruckInnsbruck, Austria; 2 Sobell Department of Motor Neuroscience and Movement Disorders, Institute of Neurology, University College London (UCL)London, United Kingdom; 3 Department of Neurology, Division of Special Neurology, Medical University of GrazGraz, Austria; 4 Reta Lila Weston Institute of Neurological Studies, UCL, Institute of NeurologyLondon, United Kingdom; 5 Institute of Nuclear Medicine, UCLLondon, United Kingdom

**Keywords:** transcranial sonography, SWEDD, Parkinson's disease, hyperechogenicity

## Abstract

**Background:**

Approximately 10% of patients clinically diagnosed with early Parkinson's disease (PD) subsequently have normal dopaminergic functional imaging. Transcranial sonography (TCS) has been shown to detect midbrain hyperechogenicity in approximately 90% of Parkinson's disease (PD) patients and 10% of the healthy population. The aim of this study was to investigate the prevalence of midbrain hyperechogenicity in patients with suspected parkinsonism and scans without evidence of dopaminergic deficit (SWEDD), in comparison to PD patients.

**Methods:**

TCS was performed in 14 patients with SWEDD and 19 PD patients.

**Results:**

There was a significantly increased area of echogenicity in the PD group (0.24 ± 0.06 cm^2^), compared to the group of patients with SWEDD (0.13 ± 0.06 cm^2^; *P* < 0.001). One (9.1%) of these patients, compared to 14 (82.5%) of the PD patients, was found to have hyperechogenicity (*P* < 0.001).

**Conclusions:**

We conclude that TCS is useful to distinguish PD patients from patients with suspected parkinsonism and SWEDD. © 2012 Movement Disorder Society

Recent clinical trials using [^123^I]β-CIT single-photon emission computed tomography (SPECT) and [^18^F]-dopa PET as surrogate markers for disease progression have found that 5.7% to 14.7% of cases clinically diagnosed as early Parkinson's disease (PD) have normal scans (scans without evidence of dopaminergic deficit; SWEDD).^1^, ^2^ In a European multicenter prospective study including diagnostically uncertain cases and performing FP-CIT-SPECT, the rate of SWEDD was 21%.^3^ The proportion of SWEDD in general practice is currently unknown, and the significance of normal imaging in patients with a clinical diagnosis of PD is still debated. There is growing evidence that these patients suffer from conditions not affecting the nigrostriatal dopaminergic system and may therefore have different pathophysiology, prognosis, and treatment requirements. SWEDD subjects in the ELLDOPA study lacked clinical responsiveness to levodopa,^4^ and follow-up dopamine transporter (DAT) scans after 4 years remained normal.^5^ Recently, it was shown that abnormality in cortical plasticity, assessed by paired associative stimulation, was markedly different in PD and tremulous SWEDD.^6^ Alternative diagnoses have been considered as essential tremor (ET), depression, vascular or psychogenic parkinsonism, dopa-responsive dystonia, supranigral parkinsonism,^4^ and primary adult-onset dystonic tremor.^7^–^9^

Certain clinical features point toward a diagnosis of tremulous SWEDD, such as lack of true bradykinesia and presence of dystonia or head tremor, whereas reemergent tremor, true fatiguing or decrement, good response to dopaminergic drugs, and the presence of nonmotor symptoms favor a diagnosis of PD.^6^, ^10^, ^11^ However, clinical distinction of tremulous SWEDD from PD remains difficult in some cases.^12^

Hyperechogenic alterations in the area of the midbrain have been consistently found in up to 90% of patients with PD in a variety of studies using transcranial sonography (TCS).^13^–^15^ Midbrain hyperechogenicity has also been observed in approximately 10% of the healthy population. Recent studies have suggested an increased risk of developing PD in a subgroup of people with midbrain hyperechogenicity.^16^, ^17^ In addition, midbrain hyperechogenicity has been associated with the development of PD in patients with idiopathic REM sleep behavior disorder.^18^, ^19^

In the present study, we aimed to investigate the prevalence of midbrain hyperechogenicity in SWEDD, in comparison to patients with PD.

## Patients and Methods

The study received approval from the local ethics committees and conformed to the Declaration of Helsinki. All participants gave written informed consent.

### Subjects

Of a total of 34 SWEDD patients observed between April 2007 and September 2009 in two movement disorder centers, 14 agreed to participate in the present study (8 from the Department of Neurology, University College London [UCL; London, UK] and 6 from the Department of Neurology, Medical University Innsbruck [Innsbruck, Austria]). All had a clinical suspected diagnosis of PD made by a neurologist and a subsequent normal DAT-SPECT scan ([^123^I]FP-CIT: *n* = 11; [^123^I]ß-CIT: *n* = 3). We additionally recruited 19 consecutive patients with a diagnosis of PD according to UK Brain Bank Criteria and abnormal [^123^I]FP-CIT SPECT scans. Striatal FP-CIT uptake was quantified by measuring the striatal/posterior lobe binding. Striatal binding of [^123^I]FP-CIT (putaminal binding of [^123^I]ß-CIT) was considered normal when the absolute tracer accumulation was >2.5 (>7.8) or the side-to-side difference of [^123^I]FP-CIT was >0.15. In both centers, patients with SWEDD had normal scans, as defined by the criteria above.^20^, ^21^

Demographic and clinical data of patients are summarized in Table 1.

**Table 1:** Demographic data, clinical data, TCS results of study patients, and diagnostic accuracy of midbrain hyperechogenicity for the differential diagnosis of PD versus SWEDD

Demographic and Clinical Data	PD Patients	SWEDD Patients	PD Versus SWEDD
Patients, n	19	14	
Female/male, n	7:12	7:7	0.497
Age, years (mean ± SD)	63.3. ± 7.5	68.2 ± 10.8	0.130
Age at onset, years (mean ± SD)	55.4 ± 11.8	56.3 ± 11.8	0.830
Disease duration, years (mean ± SD)	7.5 ± 7.2	14.9 ± 11.3	0.030*
DAT-SPECT after disease onset, years (mean ± SD)	7.2 ± 9.3	7.5 ± 7.9	0.900
Sufficient bone window, n (%)	17 (89.5)	11 (78.5)	0.630
Midbrain hyperechogenicity, n (%)	14 (82.5)	1 (9.1)	<0.001*
UPDRS motor score, mean ± SD	18 ± 10	10.8 ± 3.7	0.020*
Sensitivity, %			82.4 (58.2–94.6)
Specificity, %			90.9 (60.1–99.9)
PPV, %			93.3 (68.2–99.9)
NPV, %			76.9 (49.1–92.5)
Overall accuracy, %			85.7 (67.9–94.9)
Likelihood ratio			9.06
TP, n			14
FP, n			1
FN, n			3
TN, n			10

Data in parentheses are the confidence intervals.

* Significance level: *P* < 0.05.

Abbreviations: TP, true positive; FP, false positive; FN, false negative; TN, true negative.

### Sonography

TCS was performed using a 2.5-MHz transducer (Logiq 7; General Electric, Milwaukee, WI) from both sides using the acoustic temporal bone window. All SWEDD and PD patients were examined by the same experienced sonographer (H.S.) who was blinded to the diagnosis. TCS was performed in a random order and patients were lying in the supine position, covered with a blanket up to the neck, before the blinded investigator entered the darkened room. For analysis, only subjects were included for whom the typical butterfly-shaped mesencephalon was clearly displayed. Images of TCS examinations were stored digitally and were used for the segmentation procedure, as described previously.^13^, ^18^

To compare areas of echogenicity and the prevalence of hyperechogenicity between groups, the side of the midbrain with the greater area of echogenicity (right or left) of each subject was used for statistical comparison between groups. Hyperechogenicity of the area of SN was defined as an area of echogenic signal of 0.20 cm^2^ or greater on at least one side. This value corresponds to the 90th percentile of the area of echogenicity in the SN region of a population-based cohort of healthy individuals 50 years of age and above, who had been investigated by the same TCS examiner (H.S.), as described previously.^18^

### Statistical Analysis

Data were tabulated and analyzed using SPSS 15.0 for Windows (SPSS, Inc., Chicago, IL). Because echogenic areas of the SN were not normally distributed, as demonstrated by Shapiro-Wilks' test, Mann-Whitney's U test was applied for statistical comparisons of areas of echogenic signals in the SN between groups. Group comparison of the prevalence of hyperechogenic signal in the SN region was performed by the chi-square test for categorical variables. Between comparisons were performed with two-tailed unpaired *t* tests and Fisher's exact tests. The significance level was set at *P* < 0.05. Results are reported as means ± standard deviation (SD; median). In addition, the diagnostic accuracy of midbrain hyperechogenicity for the diagnosis of PD was calculated.

## Results

**FIG. 1:**
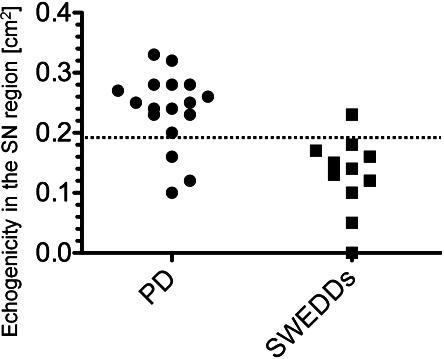
Significantly increased area of midbrain echogenicity in the PD group compared with the SWEDD group (*P* < 0.001) (broken line: value set at 0.20 cm^2^ to define hyperechogenicity).

Seventeen (89.5%) PD patients and 11 (78.5%) SWEDD patients had a sufficient acoustic temporal bone window for TCS investigation. No significant difference of area of echogenic signal was found between male and female subjects in either group. There was a significantly increased area of echogenicity in the SN region in the PD group, compared to the SWEDD group (*P* < 0.001). Mean size of echogenic area of SWEDD patients was 0.13 ± 0.06 cm^2^ (median, 0.14), compared to 0.24 ± 0.06 cm^2^ (median, 0.25) in patients with PD (*P* < 0.001) (see Fig. 1). One SWEDD patient (9.1%) was found to have midbrain hyperechogenicity defined as an area of echogenic signal of 0.20 cm^2^ or greater on at least one side, as compared to 14 (82.5%) of the PD patients (*P* < 0.001).

For diagnostic accuracy of midbrain hyperechogenicity for the differential diagnosis of PD versus SWEDD, see Table 1.

## Discussion

The clinical diagnosis of PD can be challenging in the early stage and when patients present with subtle or ambiguous signs.

In a number of studies, midbrain hyperechogenicity in the area of the SN has been a consistent finding in up to 90% of patients with idiopathic PD.^13^–^15^ A recent prospective study on a cohort of patients with different types of parkinsonism has suggested a sensitivity of 90.7% and a specificity of 82.4% of midbrain hyperechogenicity on TCS for a diagnosis of idiopathic PD (iPD).^22^

In this study, only 1 (9.1%) of the SWEDD patients was found to have midbrain hyperechogenicity. In our PD group, we detected midbrain hyperechogenicity in 82.5%. This is in line with previous studies that reported midbrain hyperechogenicity in PD patients.^13^ The overall diagnostic accuracy of TCS in distinguishing PD from SWEDD was 85%.

Observer bias is unlikely to explain these different rates, because measurements of echogenicity were performed by an experienced sonographer, who was not involved in the clinical care or assessment of the patients and was kept blinded for their imaging findings as well as for their clinical presentation. Furthermore, SWEDD patients entered the study because of the presence of clinical signs suggestive of PD, making the distinction between the groups during a brief examination with the patient lying supine very unlikely.

The present study provides further evidence that most SWEDD patients do not have PD because their midbrain echogenicity was in the same range as in control subjects, and therefore SWEDDs and PD most likely have a different underlying pathophysiology. The normal DAT-SPECT scans in patients with suspected PD prompted us to seek for alternative diagnoses, and on follow-up, new working diagnoses included adult-onset dystonic tremor (n = 6), essential tremor (n = 4), psychogenic parkinsonism (n = 2), atypical tremor (n = 1), and vascular parkinsonism (n = 1).

Recent studies found that the extent of hyperechogenicity did not correlate with the degeneration of presynaptic dopaminergic nerve terminals in patients with PD, concluding that hyperechogenicity and degeneration of presynaptic dopaminergic nerve terminals exist independently from each other and may reflect different pathomechanisms.^20^, ^23^ Midbrain hyperechogenicity in PD seems to be a stable marker that does not change in the course of the disease.^24^

TCS is a commonly available, inexpensive, and noninvasive method without exposure to radiation, but its application may be limited because of an inadequate acoustic temporal bone window, particularly in older subjects. In this study, 15.2% of subjects did not have a sufficient bone window. This is in line with previous studies showing that in approximately 15% of subjects, midbrain structures are not assessable by TCS.^13^, ^25^

In the present study, the sensitivity of midbrain hyperechogenicity on TCS for a diagnosis of iPD was 83% and the specificity was 90.9%, respectively. In patients with suspected PD, hyperechogenicity indicates a high probability of PD, with a positive predictive value (PPV) of 93.3%.

The negative predictive value (NPV) for the diagnosis of SWEDD was 76.9%, indicating that absence of hyperechogenicity would strengthen the indication for DAT imaging.

## Conclusions

We conclude that TCS is useful to distinguish patients with PD from patients with SWEDD and can provide additional information in patients presenting with inconclusive parkinsonian symptoms.

## Author Roles

(1) Research Project: A. Conception, B. Organization, C. Execution; (2) Statistical Analysis: A. Design, B. Execution, C. Review and Critique; (3) Manuscript: A. Writing of the First Draft, B. Review and Critique.

H.S.: 1A, 1B, 1C, 2A, 2C, 3A

P.S.: 1A, 1B, 1C, 3A, 3B

A.D.: 1B, 1C, 3B

L.S.-M.: 1B, 1C, 3B

P.K.: 1B, 1C, 3B

K.S.: 1A, 1C, 2A, 2B, 3B

J.D.: 1C, 3B

M.J.E.: 1A, 1B, 3B

A.J.L.: 1A, 1B, 2C, 3B

W.P.: 1A, 1B, 1C, 2C, 3B

K.P.B.: 1A, 1B, 1C, 3B

**Financial Disclosures:** P.S. received speaker honoraria from Boehringer Ingelheim and Novartis and funding for travel to congresses from Boehringer Ingelheim, Ipsen, GlaxoSmithKline (GSK), Novartis, UCB, and Merck. A.D. has been employed by UCL/University College London Hospitals (UCLH). L.S.-M. has received honoraria from Teva Lundbeck; has been awarded grants from Parkinson's UK, UCB, Genus, and Abbott; and has been employed by Reta Lila Weston Trust for Medical Research, UCL. P.K. received financial support to attend meetings from Boehringer-Ingelheim, GSK, UCB, Bayer, and Novartis pharmaceutical companies. K.S. has received honoraria from AOP Orphan Pharmaceuticals AG, Boehringer Ingelheim, and Lundbeck and has been awarded grants from Medical University Innsbruck and Oesterreichische Nationalbank. M.J.E. has received honoraria from UCB; has been awarded grants from the National Institutes for Health Research Clinician Scientist Fellowship and Parkinson's UK; has been employed by UCL; and receives royalties from the Oxford University Press. A.J.L. has held a consultancy with Genus; has served on the advisory boards of Novartis, Teva, Meda, Boehringer Ingelheim, GSK, Ipsen, Lundbeck, Allergan, Orion, BIAL, Noscira, and Roche; has received honoraria from Novartis, Teva, Meda, Boehringer Ingelheim, GSK, Ipsen, Lundbeck, Allergan, Orion, BIAL, Noscira, and Roche; has been awarded grants from PSP Association, Weston Trust-The Reta Lila Howard Foundation; and has been employed by UCL/UCLH. W.P. has received consultancy and lecture fees from AstraZeneca, Teva, Novartis, GSK, Boehringer Ingelheim, UCB, Orion Pharma, Merck Serono, and Solvay-Abbott in relation to clinical drug development programs for PD; has been awarded an AstraZeneca grant (2009–2012); and has received royalties from Oxford University press and Wiley-Blackwell. K.P.B. received funding for travel from GSK, Orion Corporation, Ipsen, and Merz Pharmaceuticals, LLC; serves on the editorial boards of Movement Disorders and Therapeutic Advances in Neurological Disorders; received royalties from Oxford University Press; received speaker honoraria from GSK, Ipsen, Merz Pharmaceuticals, LLC, and Sun Pharmaceutical Industries Ltd.; received research support from Ipsen and from the Halley Stewart Trust through Dystonia Society UK as well as the Wellcome Trust MRC strategic neurodegenerative disease initiative award (ref. no.: WT089698).

## References

[1] Whone AL, Watts RL, Stoessl AJ (2003). Slower progression of Parkinson's disease with ropinirole versus levodopa: the REALPET study. Ann Neurol.

[2] Marek K, Seibyl J (2003). Beta-CIT scans without evidemce of dopaminergic deficit (SWEDD) in the ELLDOPA-CIT and CALM-cit study: long-term imaging assessment. Neurology.

[3] Marshall VL, Reininger CB, Marquardt M (2009). Parkinson's disease is overdiagnosed clinically at baseline in diagnostically uncertain cases: a 3-year European multicenter study with repeat [123I]FP-CIT SPECT. Mov Disord.

[4] Fahn S, Oakes D, Shoulson I, Parkinson Study Group (2004). Levodopa and the progression of Parkinson's disease. N Engl J Med.

[5] Marek K, Jennings D, Seibyl J (2005). Long-term follow-up of patients with scans without evidence of dopaminergic deficit (SWEDD) in the ELLDOPA study. Neurology.

[6] Schwingenschuh P, Ruge D, Edwards MJ (2010). Distinguishing SWEDDs patients with asymmetric resting tremor from Parkinson's disease: a clinical and electrophysiological study. Mov Disord.

[7] Schneider SA, Edwards MJ, Mir P (2007). Patients with adultonset dystonic tremor resembling parkinsonian tremor have scans without evidence of dopaminergic deficit (SWEDDs). Mov Disord.

[8] Bain PG (2009). Dystonic tremor presenting as parkinsonism: long-term follow-up of SWEDDs. Neurology.

[9] Newman EJ, Breen K, Patterson J, Hadley DM, Grosset KA, Grosset DG (2009). Accuracy of Parkinson's disease diagnosis in 610 general practice patients in the West of Scotland. Mov Disord.

[10] Silveira-Moriyama L, Schwingenschuh P, O'Donnell A (2009). Olfaction in patients with suspected parkinsonism and scans without evidence of dopaminergic deficit (SWEDDs). J Neurol Neurosurg Psychiatry.

[11] Mian OS, Schneider SA, Schwingenschuh P, Bhatia KP, Day BL (2011). Gait in SWEDDs patients: comparison with Parkinson's disease and healthy controls. Mov Disord.

[12] Bajaj NP, Gontu V, Birchall J, Patterson J, Grosset DG, Lees AJ (2010). Accuracy of clinical diagnosis in tremulous parkinsonian patients: a blinded video study. J Neurol Neurosurg Psychiatry.

[13] Berg D, Godau J, Walter U (2008). Transcranial sonography in movement disorders. Lancet Neurol.

[14] Becker G, Seufert J, Bogdahn U, Reichmann H, Reiners K (1995). Degeneration of substantia nigra in chronic Parkinson's disease visualized by transcranial color-coded real time sonography. Neurology.

[15] Berg D, Siefker C, Becker G (2001). Echogenicity of the substantia nigra in Parkinson's disease and its relation to clinical findings. J Neurol.

[16] Berg D, Becker G, Zeiler B (1999). Vulnerability of the nigrostriatal system as detected by transcranial ultrasound. Neurology.

[17] Berg D, Seppi K, Behnke S (2011). Enlarged substantia nigra hyperechogenicity and risk for Parkinson disease: a 37-month 3-center study of 1847 older persons. Arch Neurol.

[18] Stockner H, Iranzo A, Seppi K, SINBAR (Sleep Innsbruck Barcelona) Group (2009). Midbrain hyperechogenicity in idiopathic REM sleep behavior disorder. Mov Disord.

[19] Iranzo A, Lomeña F, Stockner H (2010). Decreased striatal dopamine transporter uptake and substantia nigra hyperechogenicity as risk markers of synucleinopathy in patients with idiopathic rapid-eye-movement sleep behaviour disorder: a prospective study. Lancet Neurol.

[20] Spiegel J, Hellwig D, Möllers MO (2006). Transcranial sonography and [123I]FP-CIT SPECT disclose complementary aspects of Parkinson's disease. Brain.

[21] Seppi K, Scherfler C, Donnemiller E (2006). Topography of dopamine transporter availability in progressive supranuclear palsy: a voxelwise [123I]beta-CIT SPECT analysis. Arch Neurol.

[22] Gaenslen A, Unmuth B, Godau J (2008). The specificity and sensitivity of transcranial ultrasound in the differential diagnosis of Parkinson's disease: a prospective blinded study. Lancet Neurol.

[23] Doepp F, Plotkin M, Siegel L (2008). Brain parenchyma sonography and 123I-FP-CIT SPECT in Parkinson's disease and essential tremor. Mov Disord.

[24] Berg D, Merz B, Reiners K, Naumann M, Becker G (2005). Five-year follow-up study of hyperechogenicity of the substantia nigra in Parkinson's disease. Mov Disord.

[25] Stockner H, Seppi K, Kiechl S (2007). Assessment of the feasibility of midbrain sonography in a population-based study. Mov Disord.

